# Biventricular pacemaker therapy improves exercise capacity in patients with non‐obstructive hypertrophic cardiomyopathy via augmented diastolic filling on exercise

**DOI:** 10.1002/ejhf.1722

**Published:** 2020-01-23

**Authors:** Ibrar Ahmed, Brodie L. Loudon, Khalid Abozguia, Donnie Cameron, Ganesh N. Shivu, Thanh T. Phan, Abdul Maher, Berthold Stegemann, Anthony Chow, Howard Marshall, Peter Nightingale, Francisco Leyva, Vassilios S. Vassiliou, William J. McKenna, Perry Elliott, Michael P. Frenneaux

**Affiliations:** ^1^ Department of Cardiovascular Medicine University of Birmingham Birmingham UK; ^2^ Norwich Medical School University of East Anglia Norwich UK; ^3^ Lancashire Cardiac Centre Blackpool Victoria Hospital Blackpool UK; ^4^ Cardiology Department Royal Stoke University Hospital UHNM NHS Trust Newcastle UK; ^5^ Bakken Research Centre Medtronic Inc. Maastricht The Netherlands; ^6^ Department of Cardiovascular Medicine Royal Berkshire NHS Foundation Trust Reading UK; ^7^ Queen Elizabeth Hospital Birmingham Welcome Trust Clinical Research Facility Birmingham UK; ^8^ Department of Cardiovascular Medicine Queen Elizabeth Hospital Birmingham UK; ^9^ Institute of Cardiovascular Science, University College of London London UK

**Keywords:** Hypertrophic cardiomyopathy, Biventricular pacemaker therapy, Diastolic ventricular interaction

## Abstract

**Aims:**

Treatment options for patients with non‐obstructive hypertrophic cardiomyopathy (HCM) are limited. We sought to determine whether biventricular (BiV) pacing improves exercise capacity in HCM patients, and whether this is via augmented diastolic filling.

**Methods and results:**

Thirty‐one patients with symptomatic non‐obstructive HCM were enrolled. Following device implantation, patients underwent detailed assessment of exercise diastolic filling using radionuclide ventriculography in BiV and sham pacing modes. Patients then entered an 8‐month crossover study of BiV and sham pacing in random order, to assess the effect on exercise capacity [peak oxygen consumption (VO_2_)]. Patients were grouped on pre‐specified analysis according to whether left ventricular end‐diastolic volume increased (+LVEDV) or was unchanged/decreased (–LVEDV) with exercise at baseline. Twenty‐nine patients (20 male, mean age 55 years) completed the study. There were 14 +LVEDV patients and 15 –LVEDV patients. Baseline peak VO_2_ was lower in –LVEDV patients vs. +LVEDV patients (16.2 ± 0.9 vs. 19.9 ± 1.1 mL/kg/min, *P* = 0.04). BiV pacing significantly increased exercise ΔLVEDV (*P* = 0.004) and Δstroke volume (*P* = 0.008) in –LVEDV patients, but not in +LVEDV patients. Left ventricular ejection fraction and end‐systolic elastance did not increase with BiV pacing in either group. This translated into significantly greater improvements in exercise capacity (peak VO_2_ + 1.4 mL/kg/min, *P* = 0.03) and quality of life scores (*P* = 0.02) in –LVEDV patients during the crossover study. There was no effect on left ventricular mechanical dyssynchrony in either group.

**Conclusion:**

Symptomatic patients with non‐obstructive HCM may benefit from BiV pacing via augmentation of diastolic filling on exercise rather than contractile improvement. This may be due to relief of diastolic ventricular interaction.

Clinical Trial Registration: ClinicalTrials.gov NCT00504647.

## Introduction

Hypertrophic cardiomyopathy (HCM) is a common inherited disease affecting approximately 1 in 500 of the general population.^1^ Patients frequently complain of exertional breathlessness and exercise intolerance. Currently, there are effective therapies for patients in whom symptoms are due to left ventricular (LV) outflow tract obstruction.^2^ However, many symptomatic patients have no LV outflow tract obstruction at rest or on exercise, and exercise impairment appears instead to be a consequence of impaired LV diastolic filling.^3^, ^4^, ^5^ In these patients, treatment with high‐dose calcium channel blockers, beta‐blockers and diuretics is often unsuccessful.^6^

Biventricular (BiV) pacing is an effective therapy for patients with severe systolic heart failure who have associated left bundle branch block.^7^ The mechanism of improvement with BiV pacing is thought to relate to an amelioration of intraventricular contractile dyssynchrony.^8^, ^9^, ^10^ However, we have shown that some of the acute haemodynamic benefit seen with BiV pacing in chronic heart failure is due to a reduction in the external constraint to LV filling by the pericardium (pericardial constraint) and by the right ventricle through the interventricular septum [diastolic ventricular interaction (DVI)].^11^ Normally, pericardial and right ventricular end‐diastolic pressures are close to zero. Pericardial constraint and DVI occur when the pericardium becomes stretched and the pericardial and right ventricular end‐diastolic pressures become markedly increased.^12^, ^13^ We previously demonstrated that approximately 40% of patients with systolic heart failure had evidence of marked DVI at rest, and that this was predicted by a LV end‐diastolic pressure > 15 mmHg.^14^

**Figure 1:**
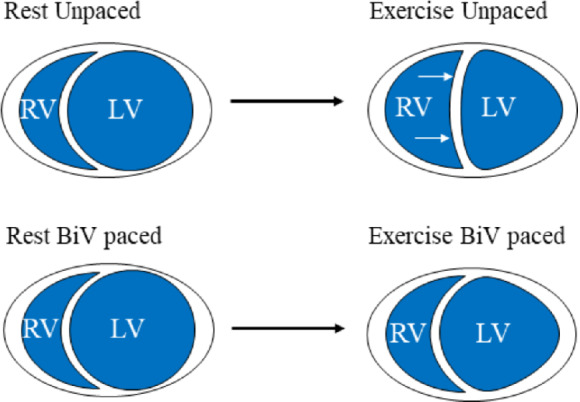
Relief of diastolic ventricular interaction during exercise with biventricular (BiV) pacing in patients with non‐obstructive hypertrophic cardiomyopathy. In patients with symptomatic non‐obstructive hypertrophic cardiomyopathy who failed to increase left ventricular end‐diastolic volume on exercise, BiV pacing corrected the left ventricular end‐diastolic volume response and improved stroke volume augmentation via the Frank–Starling mechanism, likely through relief of diastolic ventricular interaction. LV, left ventricle; RV, right ventricle.

While relatively few patients with HCM have moderate or severe pulmonary hypertension at rest, pulmonary artery pressure often rises markedly on exercise.^15^ This might be expected to cause enlargement of the right ventricle on exercise, resulting in pericardial constraint and DVI, thereby attenuating an increase in stroke volume via the Frank–Starling mechanism (*Figure* 1). In a previous study we showed that both BiV and LV pacing relieved DVI and restored the ability to use the Frank–Starling mechanism to increase stroke volume in patients with systolic heart failure.^11^ We reasoned that if DVI develops in some patients with HCM on exercise, this might be ameliorated by BiV pacing, restoring the Frank–Starling mechanism.

We therefore hypothesized that BiV pacing could improve exercise capacity in patients with non‐obstructive HCM by augmenting the Frank–Starling mechanism on exercise via enhanced diastolic filling rather than via a contractile mechanism.

## Methods

### Study design

**Figure 2:**
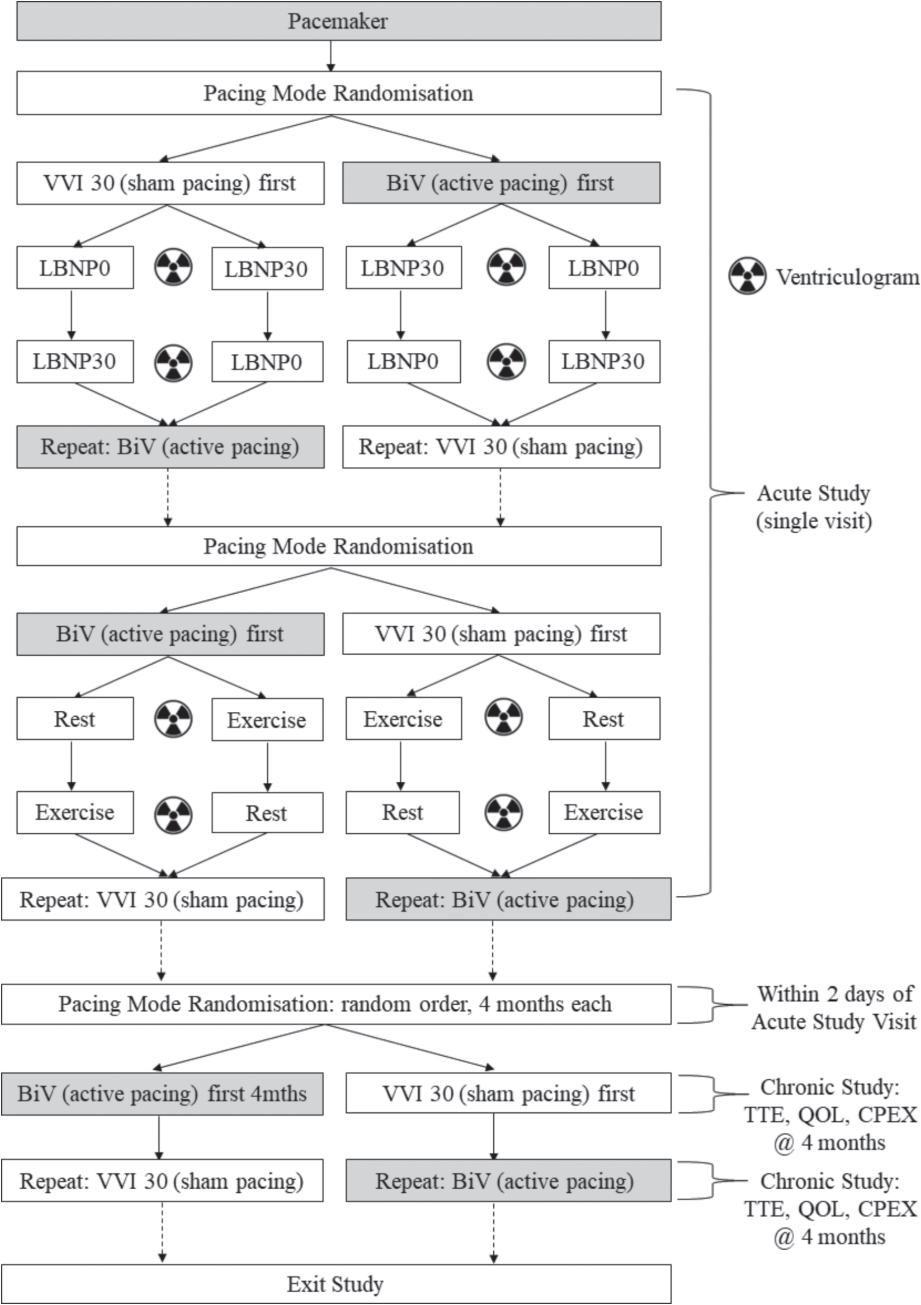
Study protocol. Following pacemaker implantation, patients were invited to attend the acute study visit, which involved radionuclide ventriculography with applied lower body negative pressures of 0 mmHg (LBNP0) and 30 mmHg (LBNP30) in the first pacing mode setting [VVI 30 or biventricular (BiV)]. This was then repeated in the second pacing mode setting. The entire protocol was then repeated at rest and on submaximal exercise to complete the acute study. Patients were then randomized into the subsequent chronic study, following a baseline transthoracic echocardiogram (TTE). CPEX, cardiopulmonary exercise test; QOL, quality of life questionnaire.

We conducted a double‐blind, randomized, crossover proof‐of‐concept study to compare the effects of BiV pacemaker therapy with sham pacing, comprising acute and chronic phases (ClinicalTrials.gov NCT00504647). Following successful BiV pacemaker implantation, patients underwent an acute crossover study to assess diastolic filling and contractile function at rest and during submaximal exercise using radionuclide ventriculography (*Figure* 2). Diastolic filling was also assessed with and without the application of lower body negative pressure to test for DVI at rest. The change in LV end‐diastolic volume (LVEDV) on exercise was used to assign patients to groups of those in whom LVEDV increased (+LVEDV), and those in whom it fell (–LVEDV). Following the acute study, patients were randomized into the chronic phase of the study to assess the effects of BiV pacing on exercise capacity, symptom status, and echocardiographic measures of dyssynchrony. The primary endpoint was change in peak oxygen consumption (VO_2_) on cardiopulmonary exercise testing during BiV pacing vs. sham pacing.

### Patient selection

Patients with exercise limitation due to non‐obstructive HCM were recruited from cardiomyopathy clinics at the Heart Hospital, London, UK and the Queen Elizabeth Hospital, Birmingham, UK. All patients provided written informed consent for the study, which was approved by the local research ethics committee and the UK Medicines & Healthcare Products Regulatory Agency, and conformed to the Declaration of Helsinki. Inclusion criteria were: age >18 years; peak VO_2_ < 75% of predicted for age and gender; New York Heart Association (NYHA) class ≥ II; sinus rhythm; and the absence of LV outflow tract obstruction (peak gradient < 30 mmHg) either at rest or during exercise. Patients were excluded on the basis of conventional indications for cardiac pacing, presence of epicardial coronary disease, pregnancy or planning to fall pregnant, and LV ejection fraction (LVEF) < 50%.

### Pacemaker implantation

Patients who fulfilled the entry criteria underwent implantation of a BiV pacing device, with the right ventricular electrode placed at the apex, and the LV electrode placed via the coronary sinus in a lateral position, using a standard technique.^16^, ^17^, ^18^ Following implantation, the pacemaker was left in VVI 30 mode for approximately 2 weeks until the acute studies were performed.

### Acute studies – gated blood pool radionuclide ventriculography

Diastolic filling studies were performed using equilibrium R‐wave gated blood pool scintigraphy (camera Olivetti Modulo‐M‐200ESL). Red blood cells were labelled by a modified *in vivo* technique.^19^ In brief, 10 min after intravenous injection of stannous pyrophosphate, 5 mL of blood was drawn into a heparinized syringe and incubated for 20 min with 750 MBq of technetium‐99m pertechnetate before reinjection. Lower body negative pressure was applied by asking patients to lie in a specially constructed lower body suction bed as previously described by us.^20^ Internal device pressure was measured via a transducer to achieve suction at 30 mmHg. Suction was turned off during the 0 mmHg negative pressure studies. The protocol was performed with the pacemaker programmed to VVI 30 for the sham pacing arm of the study. For BiV pacing, DDD mode was used, with an atrioventricular (AV) delay of 90 ms to ensure capture of the ventricles, with the left ventricle paced slightly earlier than the right ventricle [interventricular (VV) delay 40 ms]. AV and VV delays were not altered. The order of pacing modes (sham or BiV) was randomized. DVI at rest was inferred if LVEDV paradoxically increased with the application of lower body negative pressure, due to relief of the constraint on the left ventricle caused by the RV diastolic volume, as we have previously described.^21^

Volumetric data were analysed with Link Medical MAPS software (Sun Microsystems, Hampshire, UK). A count‐based ratio method was used to calculate accurate LV volumes,^22^ and end‐systolic volumes were calculated from end‐diastolic volume and ejection fraction. Intra‐observer variability for LV volumes was 2%, and inter‐observer variability was 4%. Intra‐observer variability for LVEF was 3% of the measured LVEF, with an inter‐observer variability of 5%.

For the acute exercise studies, patients exerted themselves at a workload that achieved 50% of estimated heart rate reserve. Three minutes of volumetric data were acquired at rest and during exercise after a 30 s period for stabilization of heart rate at the commencement of each stage. A 5 min ‘run‐in’ interval was given after each pacemaker mode selection. Again, this was performed during sham and BiV pacing modes, in random order. LV end‐systolic elastance was calculated from the ratio of end‐systolic pressure/end‐systolic volume indexed to body surface area.^23^ End‐systolic pressure was estimated as 90% of the brachial arterial systolic blood pressure, obtained non‐invasively via sphygmomanometer. A time‐activity curve for the left ventricle was used to derive filling fractions by splitting the diastolic filling phase (minimum volume to maximum volume) into equal tertiles. The proportion of filling occurring during each tertile of diastole was then expressed as a percentage of total diastolic filling.

### Chronic study

On completion of the acute studies, the device was returned to VVI 30 mode, and all patients underwent baseline assessments within 2 days (symptom status, cardiopulmonary exercise testing, and echocardiography). Patients were then randomized by an independent technician (to ensure clinician and patient blinding) to either sham or BiV pacing modes. After 4 months, patients underwent repeat assessment (blinded to results from the acute studies) then crossed over to the other arm of the study for a further 4 months.

### Cardiopulmonary exercise testing

Participants underwent symptom‐limited erect treadmill exercise testing (Schiller CS‐200 Ergo‐Spiro exercise machine) using a standard ramp protocol with simultaneous respiratory gas analysis.^24^, ^25^ Peak VO_2_ was defined as the highest VO_2_ achieved during exercise (with respiratory exchange ratio **>** 1.0) expressed in mL/kg/min. The minute ventilation/carbon dioxide production (VE/VCO_2_) slope was measured up to the anaerobic threshold.

### Quality of life/symptom severity assessment

Quality of life was assessed by completion of the Minnesota Living with Heart Failure Questionnaire^26^ at baseline, crossover, and completion of the study. In addition, symptom status (NYHA class) was determined by a single investigator (I.A.).

### Transthoracic echocardiography

Transthoracic echocardiography was performed with participants in the left lateral decubitus position using a Vivid 7 (GE Healthcare) echocardiographic machine with a 2.5 MHz transducer. Mean wall thickness was recorded, and LVEF was derived from the modified Simpson's formula.^27^ Conventional color‐coded tissue Doppler imaging (TDI) was performed to quantify LV dyssynchrony (EchoPac GE Medical systems). The extent of LV systolic dyssynchrony was calculated as the maximum time delay on TDI between peak systolic velocities of basal septal, lateral, anterior and inferior LV segments^9^ to derive the Yu index.^28^ For speckle tracking analysis, standard greyscale two‐dimensional images were acquired in the parasternal short axis view at the papillary muscle level. The standard deviation of the time to peak systolic radial strain for all six segments (SDt_6s_)^29^ was derived as a further index of global LV synchrony, which has the advantage over TDI of being direction‐independent. Diastolic dyssynchrony was quantified using the standard deviation of time to early peak diastolic velocities (Te‐SD) on TDI as previously described.^30^

### Statistical analysis

Data were analysed using SPSS version 22.0 for Windows and R version 3.2.3 (R Foundation for Statistical Computing, Vienna, Austria), and are expressed as mean ± standard error of the mean. The acute effect of BiV pacing on the Frank–Starling mechanism with exercise and lower body negative pressure, was evaluated by analysis of covariance (ANCOVA) using baseline resting values as covariates. Analysis of the effect of BiV pacing on peak VO_2_ was performed by repeated measures analysis of variance. Statistical significance was set at *P* < 0.05. The study had 80% power to detect a 1.5 mL/kg/min difference in peak VO_2_ between the interventions at a significance of 5%.

## Results

Overall, 31 patients were enrolled onto the study. Two patients discontinued at the crossover phase of the study. One of these patients became extremely symptomatic after crossing to the sham pacing arm of the study, and declined further participation. The second patient developed intractable diaphragmatic twitching and was unable to continue. Data are therefore presented for the 29 patients who completed the study. There were no deaths or other serious adverse device events during the study period. Baseline clinical characteristics and cardiopulmonary exercise test results of +LVEDV (*n* = 14) and –LVEDV (*n* = 15) patients are summarized in *Table* 1. A mix of devices including Guidant (13), Medtronic (10), and St Jude (6), were implanted.

**Table 1:** Baseline data

	+LVEDV	–LVEDV	*P*‐value
No. of patients	14	15	0.18
Male sex	11	9	
Age (years)	54 ± 2.6	55 ± 3.3	0.96
Resting heart rate (bpm)	63 ± 2.6	59 ± 2.1	0.29
Resting systolic BP (mmHg)	128 ± 4.2	131 ± 5.5	0.63
Resting diastolic BP (mmHg)	79 ± 1.9	75 ± 2.7	0.21
MLWHF Questionnaire score	49 ± 5.5	48 ± 6.8	0.91
QRS duration (ms)	108 ± 7.0	90 ± 3.1	0.02*
Echocardiography			
Mean wall thickness (mm)	17.7 ± 1.4	19.8 ± 1.1	0.28
LA volume index (mL/m^2^)	33.0 ± 2.8	37.6 ± 2.4	0.23
LV ejection fraction (%)	60.6 ± 1.8	62.6 ± 1.6	0.82
Mitral E velocity (m/s)	0.71 ± 0.02	0.75 ± 0.06	0.63
Mitral A velocity (m/s)	0.80 ± 0.03	0.63 ± 0.07	0.07
Mitral E/A ratio	0.9 ± 0.1	1.4 ± 0.2	0.03*
TDI S velocity (m/s) (ant‐lat)	0.05 ± 0.005	0.05 ± 0.006	0.85
TDI E′ velocity (m/s) (ant‐lat)	0.05 ± 0.005	0.05 ± 0.006	0.87
TDI A′ velocity (m/s) (ant‐lat)	0.05 ± 0.007	0.04 ± 0.005	0.55
E/E′ (antlat)	15.7 ± 3.2	15.2 ± 2.1	0.89
SD_t6s_ (s)	0.07 ± 0.012	0.05 ± 0.008	0.55
Yu index by TDI (s)	0.07 ± 0.01	0.07 ± 0.01	1.00
Te‐SD (s)	0.054 ± 0.011	0.044 ± 0.005	0.95
Medications (%)			
Beta‐blocker	7 (50)	8 (53)	0.80
ACE inhibitor	4 (29)	3 (20)	0.43
Calcium channel blocker	6 (43)	9 (60)	0.21
Diuretic	5 (36)	1 (7)	0.001*
Warfarin	0 (0)	3 (20)	0.08

Values are mean ± standard error of the mean.

A, late diastolic atrial filling wave; A′, late diastolic mitral annular tissue velocity in atrial filling; BP, blood pressure; E, early diastolic mitral inflow wave; E′, early diastolic mitral annular tissue velocity; LA, left atrial; LVEDV, left ventricular end‐diastolic volume; MLWHF, Minnesota Living with Heart Failure; SDt_6s_, standard deviation of the time to peak systolic radial strain for the six segments; Te‐SD, standard deviation of time to early peak diastolic velocities; TDI, tissue Doppler imaging.

* *P* < 0.05.

### Acute haemodynamic studies

#### Lower body negative pressure

Baseline LVEDV for the whole patient group was 120 ± 6.1 mL, and fell to 114 ± 6.0 mL with BiV pacing, but this was not statistically significant (*P* = 0.18; online supplementary *Table* *S1*). Application of 30 mmHg lower body negative pressure during sham pacing resulted in a fall in LVEDV (ΔLVEDV –16.6 ± 3.8%, *P* < 0.001). However, in four patients there was a paradoxical increase in LVEDV with the application of lower body negative pressure, implying the presence of substantial DVI at rest. This was reversed in three of these patients with the application of BiV pacing. Compared to sham pacing, BiV pacing did not change the LVEDV response to 30 mmHg lower body negative pressure (ΔLVEDV –21.1 ± 3.4%, *P* = 0.39). Stroke volume fell by a mean of 18.3 ± 3.8% with 30 mmHg lower body negative pressure during sham pacing (*P* < 0.001) and by 21.6 ± 4.1% during BiV pacing (*P* = 0.36 vs. sham pacing).

#### Exercise

**Figure 3:**
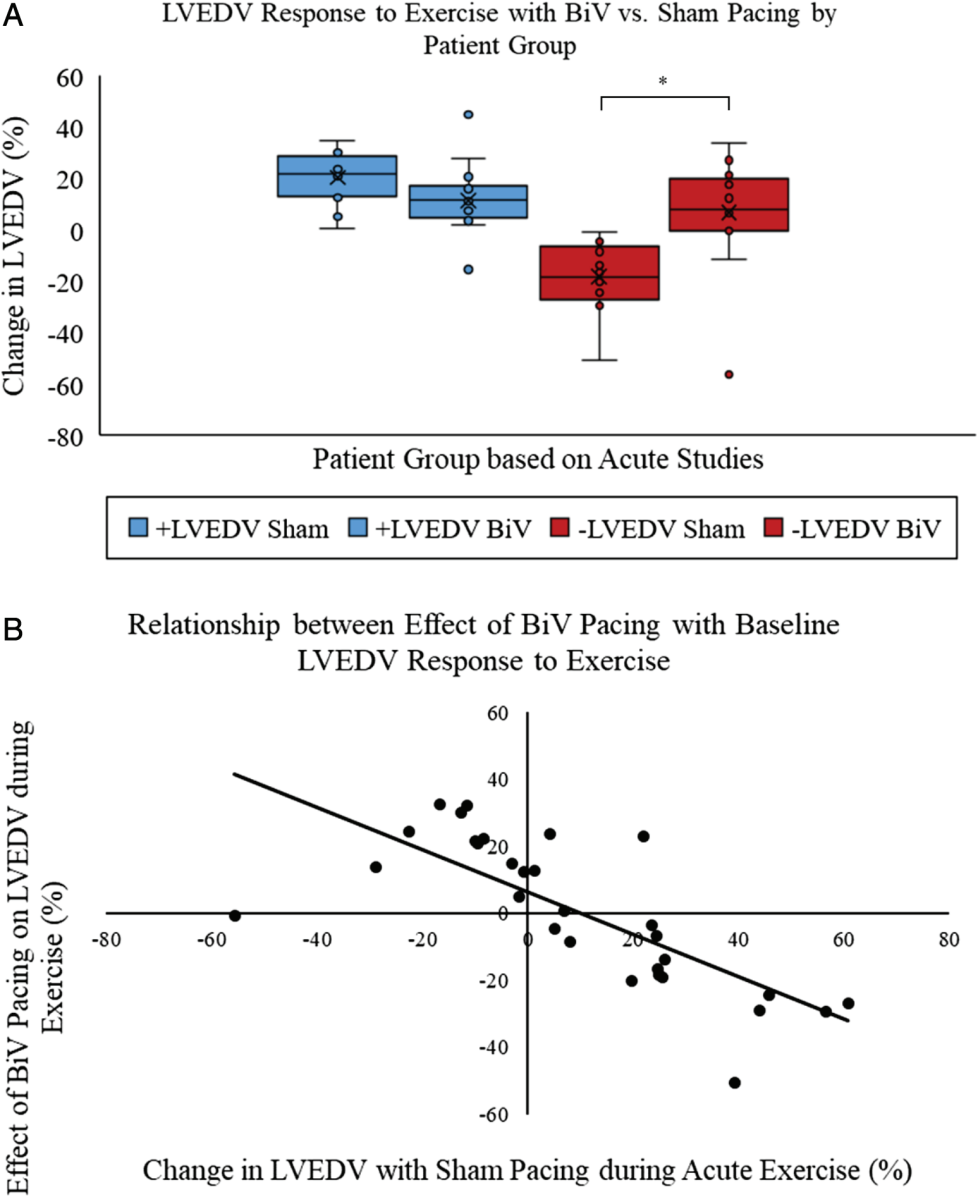
Change in left ventricular end‐diastolic volume (LVEDV) during exercise with sham and biventricular (BiV) pacing in acute exercise studies. (*A*) Patients were divided into groups based on whether LVEDV increased (+LVEDV, *n* = 14; +20.2 ± 3.8%) or fell (–LVEDV, *n* = 15; −22.3 ± 4.3%) during acute exercise testing with sham pacing (VVI 30). BiV pacing normalized the LVEDV response on exercise in –LVEDV patients (*P* = 0.004), with no effect seen in +LVEDV patients (*P* = 0.43). (*B*) There was a negative correlation in the whole patient group suggesting a relationship between ΔLVEDV% with sham pacing during acute exercise, and the effect of BiV pacing on the LVEDV response to exercise (r = −0.77, *P* < 0.001).

Patients were separated into the pre‐specified patient subgroups. +LVEDV patients (*n* = 14) had a mean increase in LVEDV on exercise of 20.2 ± 3.8% (to 141 ± 14.0 mL) during sham pacing, and –LVEDV patients (*n* = 15) experienced a mean fall of 22.3 ± 4.3% (to 106 ± 8.5 mL) (*Figure* 3 *A*). Stroke volume increased with exercise during sham pacing in +LVEDV patients by 24.4 ± 5.0% (to 111 ± 11.6 mL), and fell in –LVEDV patients by 21.0 ± 5.3% (to 71 ± 6.9 mL). With BiV pacing, –LVEDV patients demonstrated a normalization of the volume response to exercise, increasing LVEDV by 3.4 ± 7.0% (to 124 ± 8.9 mL) and stroke volume by 5.8 ± 7.6% (to 80 ± 5.2 mL), and this was significant compared to sham pacing (ΔLVEDV *P* = 0.004; ΔSV *P* = 0.008) (*Figure* 3 *A*). In contrast, in +LVEDV patients there were no significant differences in LVEDV (*P* = 0.43) or stroke volume (*P* = 0.28) responses to exercise during BiV vs. sham pacing (*Figure* 3 *A*). A significant negative correlation was seen between ΔLVEDV% on acute exercise during sham pacing, and the effect of BiV pacing on the LVEDV response to exercise (r = −0.77, *P* < 0.001) (*Figure* 3 *B*).

Left ventricular end‐systolic elastance at rest did not differ between sham pacing vs. BiV pacing in either +LVEDV patients (2.96 ± 0.5 vs. 2.59 ± 0.4 mmHg/mL, *P* = 0.57) or –LVEDV patients (2.44 ± 0.3 vs. 2.84 ± 0.4 mmHg/mL, *P* = 0.43) (*Table* 2). Similarly, left ventricular end‐systolic elastance did not differ in either group with sham vs. BiV pacing during exercise (+LVEDV: 3.15 ± 0.5 vs. 3.10 ± 0.5 mmHg/mL, *P* = 0.93; –LVEDV: 3.62 ± 0.5 vs. 3.54 ± 0.5 mmHg/mL, *P* = 0.91). LVEF did not differ between BiV pacing and sham pacing at rest (72.4 ± 2.3% vs. 73.8 ± 7.7%, *P* = 0.62), or during exercise (73.7 ± 2.4% vs. 74.1 ± 2.1%, *P* = 0.90), and there were no differences between –LVEDV and +LVEDV patients (*Table* 2). However, BiV pacing was associated with a significant shortening of the duration of systole compared to sham pacing, both at rest (0.38 ± 0.02 s vs. 0.33 ± 0.02 s, *P* = 0.03) and during exercise (0.32 ± 0.01 s vs. 0.27 ± 0.01 s, *P* = 0.002).

**Table 2:** Acute semi‐supine exercise data

Test variable	Sham	BiV	*P*‐value
**Rest (*n* = 29)**			
+LVEDV patients (*n* = 14)			
LVEF (%)	74 ± 3.0	76 ± 2.4	0.58
Heart rate (bpm)	59 ± 2.1	64 ± 2.7	0.19
Systole_dur_ (s)	0.37 ± 0.02	0.32 ± 0.02	0.13
Diastole_dur_ (s)	0.57 ± 0.05	0.59 ± 0.04	0.81
Final 2/3 filling (%)	51 ± 4.4	66 ± 3.5	0.01*
E_LV_ (mmHg/mL)	2.96 ± 0.5	2.59 ± 0.4	0.57
–LVEDV patients (*n* = 15)			
LVEF (%)	71 ± 3.4	72 ± 2.5	0.87
Heart rate (bpm)	63 ± 2.6	67 ± 3.6	0.29
Systole_dur_ (s)	0.38 ± 0.03	0.34 ± 0.02	0.14
Diastole_dur_ (s)	0.61 ± 0.05	0.56 ± 0.06	0.19
Final 2/3 filling (%)	52 ± 4.4	69 ± 4.9	0.02*
E_LV_ (mmHg/mL)	2.44 ± 0.3	2.84 ± 0.4	0.43
**Exercise (*n* = 29)**			
+LVEDV patients (*n* = 14)			
LVEF (%)	76 ± 3.6	75 ± 3.4	0.85
Heart rate (bpm)	87 ± 4.6	87 ± 4.6	0.98
Systole_dur_ (s)	0.32 ± 0.01	0.26 ± 0.02	0.05*
Diastole_dur_ (s)	0.35 ± 0.04	0.38 ± 0.04	0.47
Final 2/3 filling (%)	66 ± 3.7	76 ± 3.0	0.04*
E_LV_ (mmHg/mL)	3.15 ± 0.5	3.10 ± 0.5	0.93
–LVEDV patients (*n* = 15)			
LVEF (%)	71 ± 3.1	73 ± 2.7	0.66
Heart rate (bpm)	91 ± 3.9	93 ± 4.5	0.69
Systole_dur_ (s)	0.33 ± 0.01	0.27 ± 0.01	0.02*
Diastole_dur_ (s)	0.34 ± 0.02	0.44 ± 0.03	0.002*
Final 2/3 filling (%)	62 ± 4.3	80 ± 3.2	0.003*
E_LV_ (mmHg/mL)	3.62 ± 0.5	3.54 ± 0.5	0.91

Values are mean ± standard error of the mean.

BiV, biventricular pacing; Diastole_dur_, duration of diastole; E_LV_, left ventricular end‐systolic elastance; LVEDV, left ventricular end‐diastolic volume; LVEF, left ventricular ejection fraction; Systole_dur_, duration of systole; VVI 30, ventricular pacing and sensing at 30 bpm (sham pacing).

* *P* < 0.05.

Diastolic filling time increased on exercise with BiV vs. sham pacing in –LVEDV patients (0.34 ± 0.02 s vs. 0.44 ± 0.03 s, *P* = 0.002), but not in +LVEDV patients (0.35 ± 0.04 s vs. 0.38 ± 0.04 s, *P* = 0.47). In –LVEDV patients, the contribution of the final two thirds of diastole to LV filling during exercise increased with BiV pacing compared to sham pacing (62 ± 4.3% vs. 80 ± 3.2%, *P* = 0.003), and this was also true for +LVEDV patients (66 ± 3.7% vs. 76 ± 3.0%, *P* = 0.04) (*Table* 2).

### Chronic study

#### Exercise

**Figure 4:**
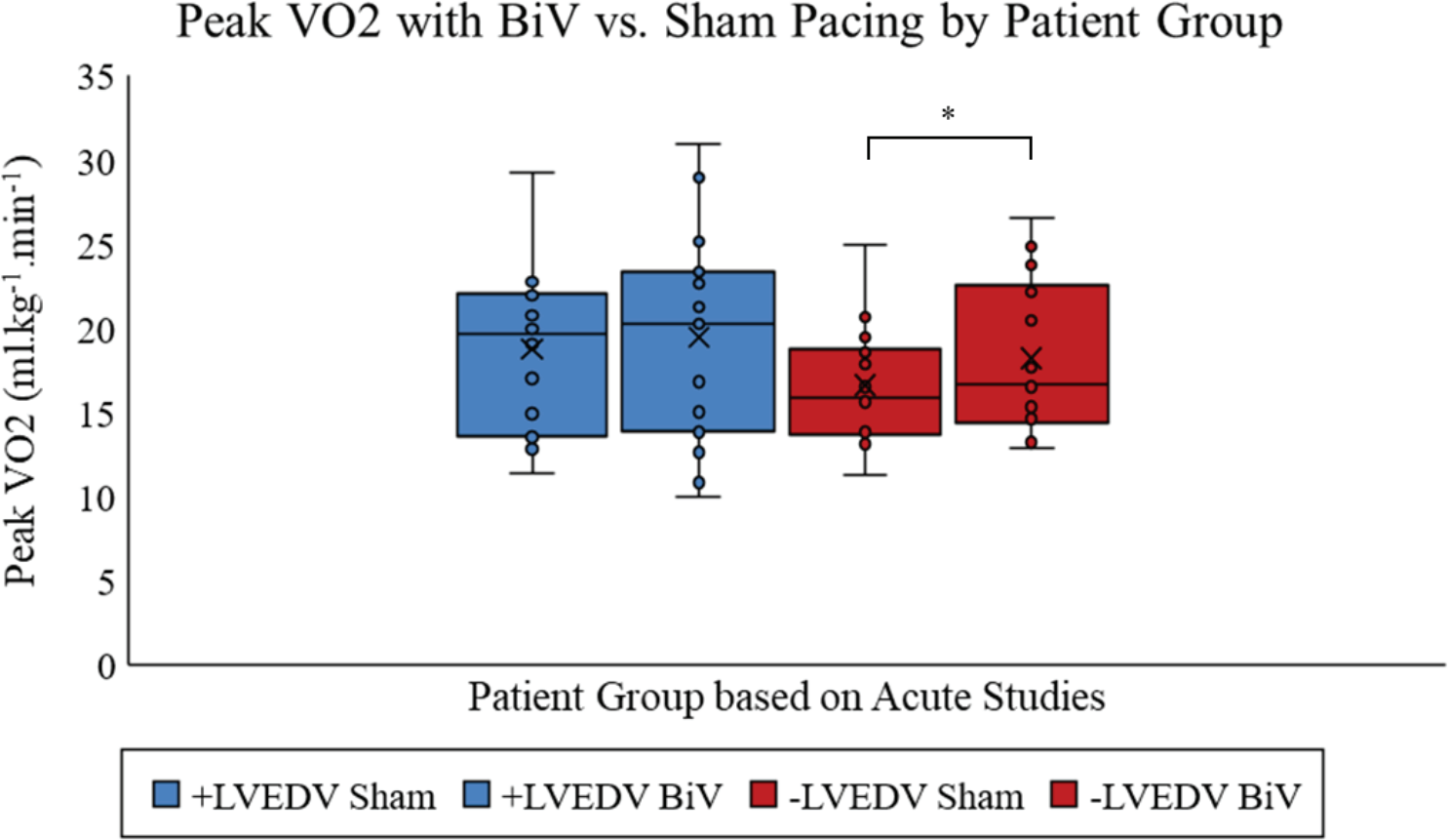
Change in peak oxygen consumption (VO_2_) during 4 months of biventricular (BiV) vs. 4 months of sham pacing by patient group. Compared to sham, BiV pacing increased peak VO_2_ in patients with decreased left ventricular end‐diastolic volume (–LVEDV) by 1.4 mL/kg/min (16.2 ± 0.9 vs. 17.6 ± 1.2 mL/kg/min) and this was statistically significant (*P* = 0.03). A small increase was seen in patients with increased LVEDV (+LVEDV) (19.9 ± 1.1 vs. 20.8 ± 1.5 mL/kg/min), but this was not statistically significant (*P* = 0.13).

Baseline peak VO_2_ was significantly lower in –LVEDV patients compared to +LVEDV (16.4 ± 0.9 vs. 19.5 ± 1.1 mL/kg/min, *P* = 0.04 vs. –LVEDV). BiV pacing increased peak VO_2_ in the whole patient group compared to sham pacing (+1.17 mL/kg/min, *P* = 0.02; online supplementary *Table* *S2*). By pre‐specified patient subgroup, peak VO_2_ increased significantly during BiV pacing vs. sham in –LVEDV patients (16.2 ± 0.9 vs. 17.6 ± 1.2 mL/kg/min, *P* = 0.03). There was a slight and non‐significant increase in +LVEDV patients (19.9 ± 1.1 vs. 20.8 ± 1.5 mL/kg/min; *P* = 0.13) (*Table* 3 and *Figure* 4).

**Table 3:** Exercise and quality of life data following 4‐month pacing intervention

Test variable	Sham	BiV	*P*‐value
**Quality of life**			
+LVEDV patients *(n* = 14)			
MLWHF Questionnaire score	45 ± 6.2	38 ± 5.5	0.05
–LVEDV patients (*n* = 15)			
MLWHF Questionnaire score	50 ± 5.0	35 ± 5.9	0.02*
**Exercise**			
+LVEDV patients (*n* = 14)			
Resting heart rate (bpm)	67 ± 3.3	67 ± 3.4	1.00
Peak heart rate (bpm)	128 ± 6.8	123 ± 6.5	0.56
Peak systolic BP (mmHg)	159 ± 6.1	168 ± 5.9	0.48
Exercise duration (s)	462 ± 28	469 ± 28	1.00
RER	1.08 ± 0.02	1.09 ± 0.02	1.00
VE/VCO_2_	36.3 ± 1.4	35.1 ± 1.4	0.82
Peak VO_2_ (mL/kg/min)	19.9 ± 1.1	20.8 ± 1.5	0.13
–LVEDV patients (*n* = 15)			
Resting heart rate (bpm)	63 ± 2.9	59 ± 2.1	0.28
Peak heart rate (bpm)	111 ± 5.8	118 ± 5.7	0.50
Peak systolic BP (mmHg)	158 ± 7.6	163 ± 6.7	1.00
Exercise duration (s)	409 ± 26	461 ± 25	0.008*
RER	1.09 ± 0.03	1.09 ± 0.02	1.00
VE/VCO_2_	34.3 ± 2.8	36.0 ± 1.6	1.00
Peak VO_2_ (mL/kg/min)	16.2 ± 0.9	17.6 ± 1.2	0.03*

Values are mean ± standard error of the mean.

BiV, biventricular pacing; BP, blood pressure; LVEDV, left ventricular end‐diastolic volume; MLWHF, Minnesota Living with Heart Failure; RER, respiratory exchange ratio; VCO_2_, carbon dioxide production; VE, minute ventilation; VO_2_, oxygen consumption.

* *P* < 0.05.

#### Quality of life

Minnesota Living with Heart Failure Questionnaire scores improved significantly with BiV pacing compared to sham pacing in the whole patient group (*P* = 0.001) (online supplementary *Table* *S2*), and this was true for the –LVEDV subgroup (*P* = 0.02), with a strong trend observed in +LVEDV patients (*P* = 0.05) (*Table* 3).

#### Left ventricular dyssynchrony

The two parameters of systolic dyssynchrony, SD_t6s_ and Yu index, did not demonstrate reduced dyssynchrony scores following 4 months of BiV pacing compared to sham (*P* = 1.00 and *P* = 0.25, respectively) (online supplementary *Table* *S2*). Te‐SD, the measure of diastolic dyssynchrony, was also similar with each pacing mode (*P* = 1.00).

## Discussion

In this study we show that BiV pacing increased exercise capacity and improved quality of life in patients with non‐obstructive HCM who have severe exercise limitation due to breathlessness despite optimal, maximally‐tolerated standard therapies. The magnitude of improvement in peak VO_2_ was comparable to that seen in cardiac resynchronization trials in systolic heart failure.^31^

In patients with systolic heart failure, the predominant mechanism of improvement of cardiac performance by BiV pacing has been considered to be due to the relief of mechanical dyssynchrony. In this study, QRS duration was normal and LVEF was at least 50% at rest in all patients. Furthermore, the degree of resting systolic mechanical dyssynchrony present in these patients was much less marked than in patients with systolic heart failure who undergo BiV pacemaker implants.^32^ No significant changes in these measures of systolic dyssynchrony (at rest) were observed with BiV pacing. Furthermore, there was no significant effect of BiV pacing on LVEF or on LV end‐systolic elastance (a relatively load‐independent measure of LV contractile function) at rest or with exercise. These findings argue against a substantial beneficial effect of BiV pacing on LV contractile function (at the ‘chamber’ level) that might explain the improved volume response on exercise and the increase in exercise capacity observed.

Importantly, BiV pacing had marked effects on the filling of the left ventricle with exercise, and this was closely related to the baseline exercise LVEDV response used to assign patient groups (*Figure* 3 *B*). In health, LVEDV increases during exercise, and thereby the Frank–Starling mechanism contributes to the increase in stroke volume.^33^ This is in part a consequence of an increase in the rate of LV active relaxation during exercise.^34^ We have previously shown that the rate of LV active relaxation paradoxically slows during exercise in many patients with HCM, and that amelioration of cardiac energetic impairment with the metabolic modulator perhexiline reverses this abnormality.^35^ Approximately 50% of the patients in this study had an abnormal fall in LVEDV during exercise (–LVEDV), and an associated fall in stroke volume. This pattern was associated with more severe exercise limitation than those in whom LVEDV increased as expected on exercise (+LVEDV). BiV pacing substantially corrected these abnormal LVEDV and stroke volume responses in –LVEDV patients, and significantly increased peak VO_2_ (by 1.4 mL/kg/min), but no significant effect on LVEDV or stroke volume during exercise was observed in +LVEDV patients. The increase in peak VO_2_ was also smaller and was not statistically significant. Thus, the improved cardiac performance on exercise and exercise capacity in –LVEDV patients is principally due to partial restoration of the ability to use the Frank–Starling mechanism to increase stroke volume with exercise.

Potential explanatory mechanisms underlying improved augmentation of diastolic filling on exercise include a reduction in intrinsic LV stiffness, an increase in diastolic filling time, or a reduction in external constraint to LV filling. Diastolic dyssynchrony has been reported in HCM^36^; however, we observed no significant effect of BiV pacing on measures of diastolic dyssynchrony at rest, and BiV pacing reduced the first‐third filling fraction at rest and on exercise (the period that includes active relaxation), which argues against this being an important mechanism. We have previously shown that external constraint to LV filling by the pericardium^37^ (pericardial constraint) and by the right ventricle through the interventricular septum (DVI) is observed in patients with chronic heart failure who have elevated LV end‐diastolic pressures (typically >15 mmHg).^14^ In a previous study of patients with severe chronic heart failure, we showed that both BiV and LV pacing reduced this external constraint to LV filling and recruited LV preload, presumably by shifting the timing of LV filling in relation to the right ventricle.^11^

In the present study we report a paradoxical increase in LVEDV during application of lower body negative pressure in four patients (all –LVEDV patients), suggesting the presence of significant DVI at rest. In three of these patients, this paradoxical increase in LVEDV during application of lower body negative pressure was normalized by BiV pacing, suggesting alleviation of DVI. Although only a minority of patients with HCM have pulmonary hypertension and markedly raised LVEDP at rest, both pulmonary artery pressure and LVEDP markedly rise during exercise in many patients with symptomatic HCM,^15^ therefore *a priori* DVI might be expected to be a frequent occurrence on exercise in these patients, by analogy with our recent findings in patients with heart failure with a preserved ejection fraction.^38^ We suggest that the partial restoration of the Frank–Starling mechanism on exercise by BiV pacing in –LVEDV patients is most likely explained by relief of DVI. DVI is typically associated with a restrictive LV filling pattern, in which rapid early filling ceases with the onset of external constraint. Of note, BiV pacing during exercise reduced the rate of early filling but markedly increased LV filling in the later part of diastole, consistent with our hypothesis. Larger randomized controlled trials are needed to confirm these findings and determine whether they translate to improved survival. Indeed, the EchoCRT study of patients with systolic heart failure and a short QRS duration was halted early by the data and safety committee for futility with a potential for harm with cardiac resynchronization therapy/BiV pacing.^39^ Whether similar adverse effects might occur in our patients is unknown; however, there were no significant adverse events or significant adverse device events during the 4‐month BiV pacing arm of our study.

### Study limitations

The HCM phenotype can be caused by over 400 genetic mutations in the sarcomeric contractile apparatus.^40^ The pattern and severity of hypertrophy and contractile dysfunction is often heterogeneous between patients and indeed between different areas of an individual patient's myocardium. However, all of our patients fulfilled the currently established criteria for HCM,^40^ and as much as possible we have excluded the presence of HCM phenocopies such as infiltrative cardiomyopathies (e.g. amyloid) and glycogen storage disorders. We have also used ‘chamber’ level measures of contractile function, reducing the impact of regional variations. Highly symptomatic non‐obstructive HCM is relatively uncommon, and identifying and recruiting appropriate patients remains difficult, despite recruitment from two large tertiary referral centres in the UK. However, the majority of randomized controlled trials in this patient subgroup involve similar patient numbers, and we attempted to maximize statistical power with a crossover study design. Despite achieving >80% power for the primary endpoint, the relatively small number of patients in our study may have important effects on the results seen. Changes in peak VO_2_ are greatly influenced by changes in heart rate,^41^ which can be quite variable in smaller sample sizes. Numerically, exercise peak heart rate was higher in +LVEDV patients than –LVEDV patients, and peak heart rate was numerically higher during BiV pacing in –LVEDV patients with no change in +LVEDV patients. This mirrored the improvement seen in peak VO_2_. It is important to note, however, that these differences were not statistically significant and the changes in cardiac volumes were much more marked. Whilst it is true that peak exercise heart rate was higher in –LVEDV patients (and slightly higher during BiV pacing than sham), BiV pacing corrected the fall in LVEDV and stroke volume in these patients. Therefore, the very large changes in LVEDV and stroke volume are much more likely to be responsible for driving the increase in exercise capacity in these patients.

We hypothesized that BiV pacing ameliorated DVI with exercise in –LVEDV patients, and that this resulted from exercise‐induced pulmonary hypertension. Unfortunately, the tricuspid regurgitant jet in the majority of patients was insufficient to allow prediction of right heart pressures at rest. Indeed, right ventricular pressures during exercise are likely more important, and would have required invasive right heart catheterization, which was not undertaken in an already complex protocol for patients. Similarly, we did not attempt to optimize AV or VV delay during BiV pacing in our patients. In patients with systolic heart failure, AV optimization has been shown to improve response to cardiac resynchronization therapy, but does not change non‐responders to responders.^42^ We also chose a specific VV delay of 40 ms from previous work demonstrating a potential benefit when the left ventricle is triggered slightly earlier than the right ventricle.^11^ AV and VV optimization of BiV pacing in non‐obstructive HCM represents an interesting topic for future studies.

## Conclusion

Biventricular pacing improved symptoms and exercise capacity in patients with non‐obstructive HCM. The benefit was greatest in those patients with the most marked diastolic impairment during exercise, and the benefit was due to augmented diastolic filling on exercise, enhancing utilization of the Frank–Starling mechanism. We suggest that the most likely mechanism of this improvement in diastolic filling is relief of DVI. Larger studies of BiV pacing in non‐obstructive HCM are indicated.

### Funding

This work was funded by British Heart Foundation grant PG/05/087 and a grant‐in‐aid from Medtronic.

**Conflict of interest:** B.S. is a full‐time employee of Medtronic. F.L. has received honoraria from Medtronic. The other authors have nothing to disclose.

## Supporting information


**Figure S1.** Left ventricular end‐diastolic volume response to acute submaximal exercise with VVI 30 (sham) and biventricular pacing.

**Table S1.** Change in left ventricular volumes during acute studies.
**Table S2.** Chronic follow‐up data for whole patient group.
