# Supplementary figures and images for: Biventricular pacemaker therapy improves exercise capacity in patients with non‐obstructive hypertrophic cardiomyopathy via augmented diastolic filling on exercise
Source: Eur J Heart Fail. 2020 Jan 23;22(7):1263–72. doi: 10.1002/ejhf.1722 (PMC7540697; doi:10.1002/ejhf.1722)

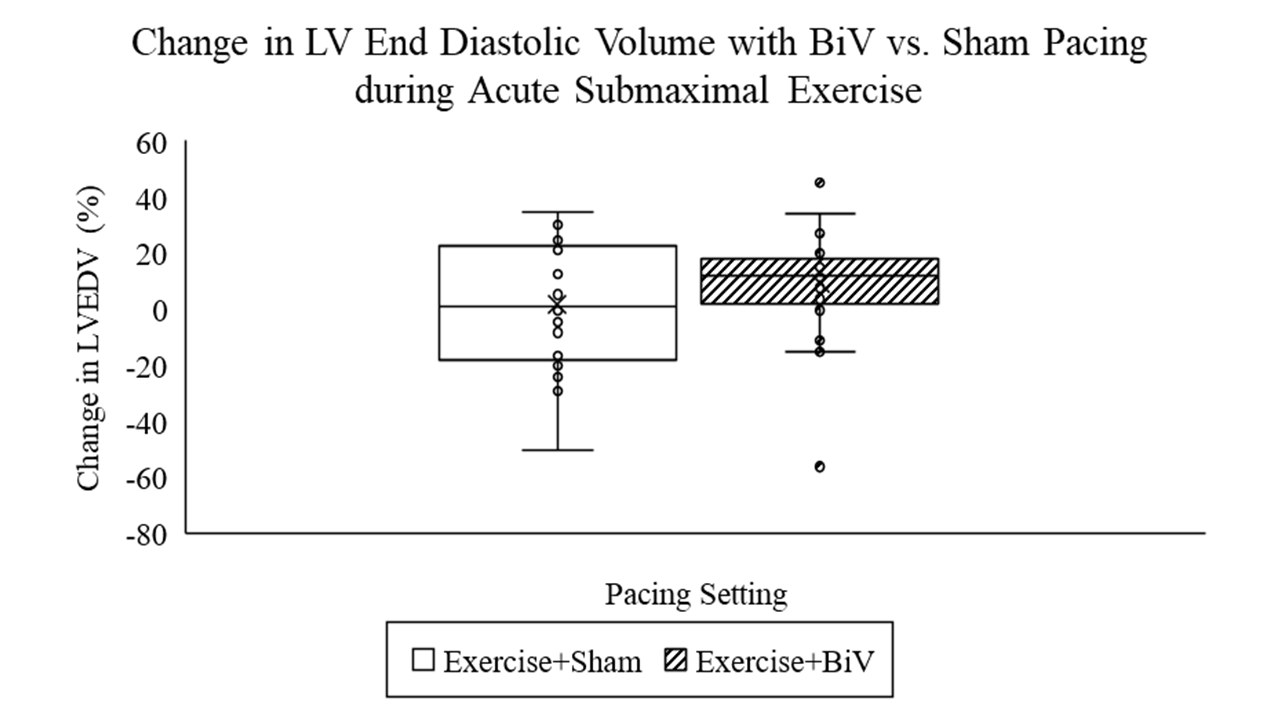

Supplement: Supplementary file 1 — Figure S1. Left ventricular end‐diastolic volume response to acute submaximal exercise with VVI 30 (sham) and biventricular pacing. [file EJHF-22-1263-s001.tif]
