# Supplementary material for: Biventricular pacemaker therapy improves exercise capacity in patients with non‐obstructive hypertrophic cardiomyopathy via augmented diastolic filling on exercise
Source: Eur J Heart Fail. 2020 Jan 23;22(7):1263–72. doi: 10.1002/ejhf.1722 (PMC7540697; doi:10.1002/ejhf.1722)
Supplement: Supplementary file 2 — Table S1. Change in left ventricular volumes during acute studies. Table S2. Chronic follow‐up data for whole patient group. [file EJHF-22-1263-s002.docx]

**Supplementary Material**

*Supplemental Table 1: Change in LV Volumes during Acute Studies*

| **Test Variable** | VVI 30 | | BiV | *p* value |
| --- | --- | --- | --- | --- |
| **Rest (n=29)** | | | | |
| LVEDV (ml) | 120 ± 6.1 | 114 ± 6.0 | | 0.18 |
| SV (ml) | 86 ± 5.3 | 81 ± 5.2 | | 0.31 |
| **Lower Body Negative Pressure (n=29)** | | | | |
| LVEDV (ml) | 97 ± 12.4 | 81 ± 6.3 | | 0.39 |
| SV (ml) | 66 ± 7.7 | 55 ± 4.1 | | 0.36 |
| **Exercise (n=29)** | | | | |
| LVEDV (ml) | 125 ± 12.2 | 122 ± 6.2 | | 0.18 |
| SV (ml) | 93 ± 7.6 | 91 ± 5.1 | | 0.31 |
| *+LVEDV Patients (n=14)* | | | | |
| LVEDV (ml) | 141 ± 14.0 | 127 ± 9.3 | | 0.43 |
| SV (ml) | 111 ± 11.6 | 100 ± 7.9 | | 0.28 |
| *–LVEDV Patients (n=15)* | | | | |
| LVEDV (ml) | 106 ± 8.5 | 124 ± 8.9 | | 0.004^*^ |
| SV (ml) | 71 ± 6.9 | 80 ± 5.2 | | 0.008^*^ |

Values are mean ± SEM. ^*^ *p* < 0.05. BiV pacing compared to sham pacing corrected the fall in LVEDV and SV on exercise in –LVEDV patients. BiV, biventricular pacing, BP, blood pressure; LVEDV, left ventricular end diastolic volume; SV, stroke volume; VVI 30, ventricular pacing and sensing at 30bpm (sham pacing).

*Supplemental Table 2: Chronic Follow-up Data for Whole Patient Group*

| **Test Variable** | **All Patients (n=29)** | | |
| --- | --- | --- | --- |
|  | VVI 30 | BiV | *p* value |
| **Quality of Life** | | | |
| Minnesota LWHF Questionnaire Score | 47 ± 4.1 | 37 ± 4.1 | 0.001^*^ |
| **LV Dyssynchrony** | | | |
| SDt_6s_ (s) | 0.05 ± 0.009 | 0.04 ± 0.007 | 1.00 |
| Yu index by TDI (s) | 0.05 ± 0.003 | 0.04 ± 0.004 | 0.25 |
| Te-SD (s) | 0.05 ± 0.008 | 0.04 ± 0.006 | 1.00 |
| **Exercise** | | | |
| Peak Heart Rate (bpm) | 120 ± 4.5 | 121 ± 4.3 | 1.00 |
| Peak Systolic BP (mmHg) | 158 ± 4.8 | 166 ± 4.4 | 0.37 |
| Exercise duration (s) | 437 ± 19 | 465 ± 18 | 0.02^*^ |
| RER | 1.09 ± 0.02 | 1.09 ± 0.02 | 1.00 |
| VE/VCO_2_ | 35.2 ± 1.6 | 35.5 ± 1.1 | 1.00 |
| VO_2_ max (% of max predicted) | 43.6 ± 1.6 | 46.6 ± 2.1 | 0.01^*^ |
| Peak VO_2_ (ml.kg^-1^.min^-1^) | 18.0 ± 0.8 | 19.2 ± 1.0 | 0.02^*^ |

Values are mean ± SEM. ^*^ *p* < 0.05. BiV pacing improved exercise capacity and quality of life scores compared to sham pacing at 4 months in symptomatic non-obstructive HCM patients. BiV, biventricular pacing; BP, blood pressure; LWHF, living with heart failure; RER, respiratory exchange ratio; SD_t6s_, standard deviation of the time to peak-systolic radial strain for all six segments; TDI, tissue Doppler imaging; Te-SD, standard deviation of time to early peak diastolic velocities; VCO_2_, carbon dioxide production; VE, minute ventilation; VO_2_, oxygen consumption; VVI 30, ventricular pacing and sensing at 30bpm (sham pacing).

*
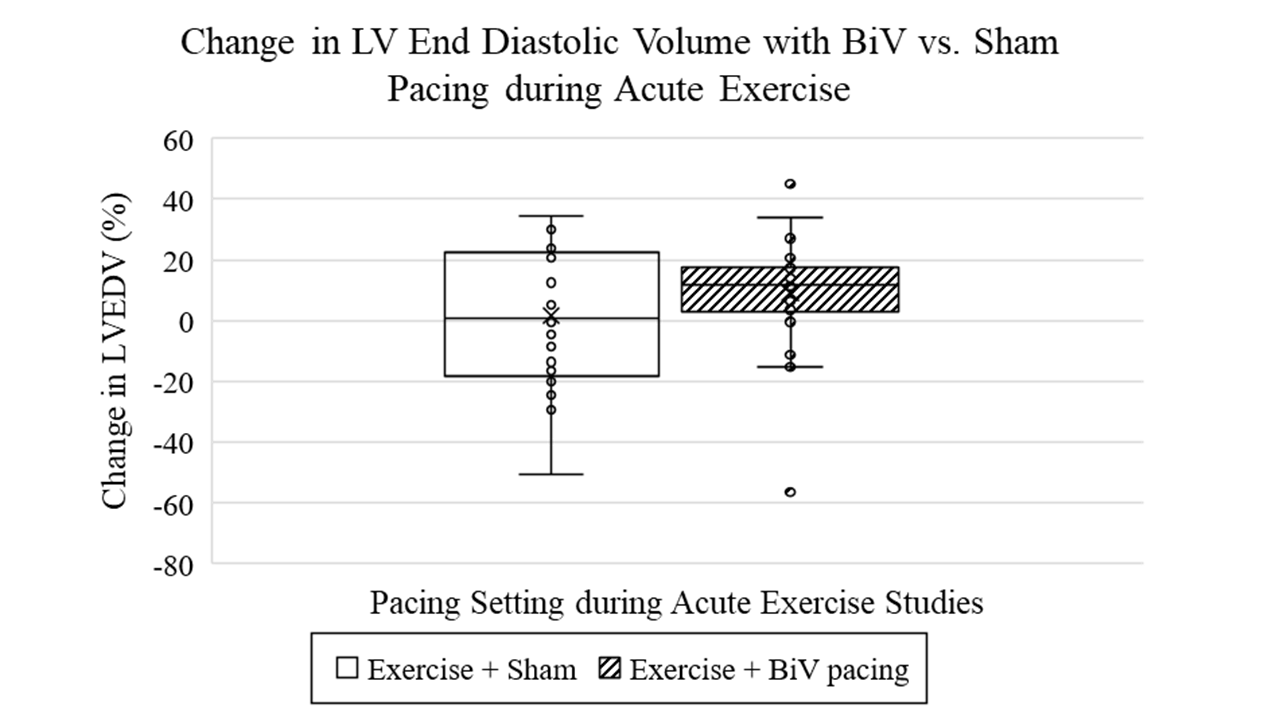
*

*Supplemental Figure 1: LVEDV Response to Acute Submaximal Exercise with VVI 30 (Sham) and BiV Pacing*

*In the whole patient group, there was a heterogeneous response to exercise, and there was a trend towards improved LVEDV response to exercise with BiV pacing, but this was not statistically significant (ΔLVEDV%, n=29; BiV vs. VVI30, p=0.18). BiV, Biventricular Pacing; LVEDV, Left Ventricular End Diastolic Volume; ΔLVEDV, change in LVEDV with exercise = LVEDV_exe_ – LVEDV_rest_; VVI 30, ventricular pacing and sensing at 30 bpm (sham pacing).*
